# Supplementary material for: Building a pipeline to identify and engineer constitutive and repressible promoters
Source: Quant Plant Biol. 2023 Oct 19;4:e12. doi: 10.1017/qpb.2023.10 (PMC10600573; doi:10.1017/qpb.2023.10)
Supplement: Yang and Nemhauser supplementary material 1 — Yang and Nemhauser supplementary material [file S2632882823000103sup001.docx]

### Supplementary materials

Supplementary Table S1. qPCR primers for RUBY and PP2AA3

| Gene | Forward Primer | Reverse Primer |
| --- | --- | --- |
| RUBY | AAACAGGGCAAGCTCGTGTA | ATCCGCAGTGGGTGAGAAAG |
| PP2AA3 | AACGTGGCCAAAATGATGC | AACCGCTTGGTCGACTATCG |

Supplementary Table S2. Top 3% Candidate Genes

AGI is the *Arabidopsis* Genome Initiative locus code. Coefficient of variation (CV) and geometric mean (geom_mean) are calculated without the stress dataset while coefficient of variation for the stress dataset (StressCV) is given separately. The core promoter types (TATA, Ypatch, CA, GA, Coreless) are taken from Tokizawa et al. 2017, and “1” signifies that core type is predicted in the promoter. CGDB circadian genes are whether the promoter is found to be circadian regulated.


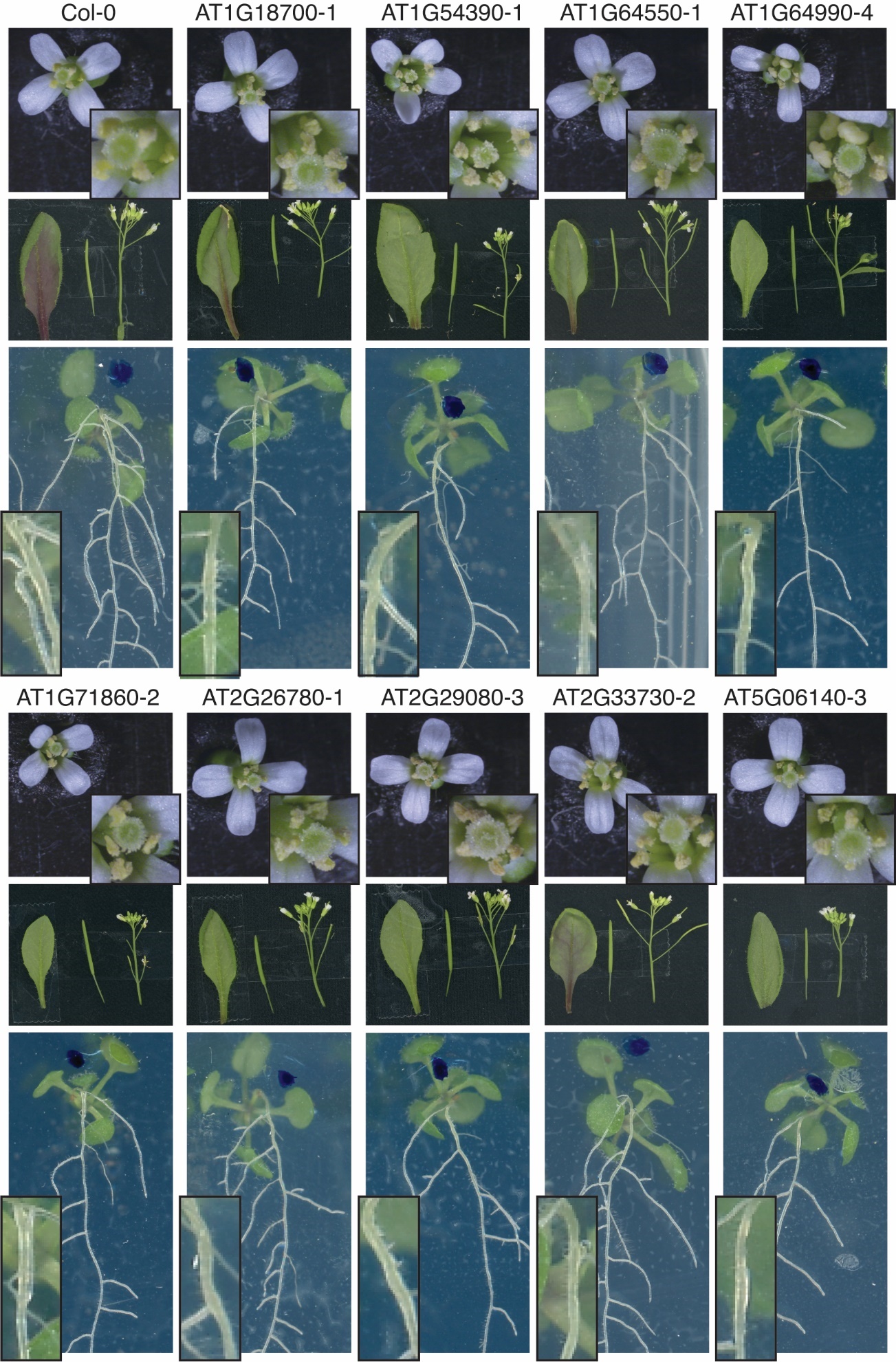


Supplementary Figure S3. *Arabidopsis* T2 plants transformed with various promoters driving reporter RUBY. The flowers, siliques, and leaves are captured on day 34 while the seedling images are captured on day 12. The inset boxes are zoomed in pictures of their associated images. Areas where there are RUBY expression visible by eye is marked by the red arrow


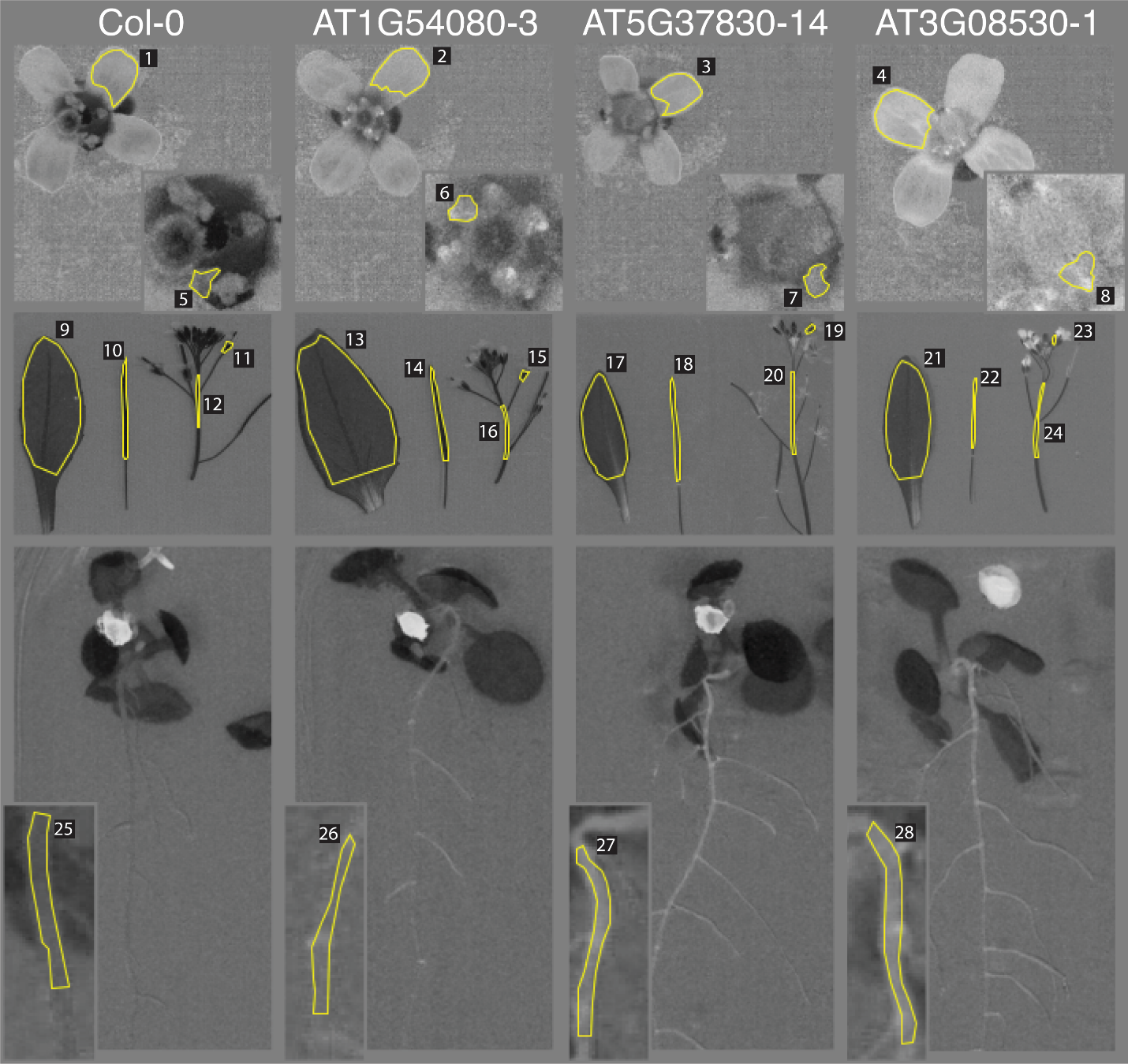


|  | Col-0 | AT1G54080-3 | AT5G37830-14 | AT3G08530-1 |
| --- | --- | --- | --- | --- |
| Petal | 1 : 148 | 2 : 160 | 3 : 158 | 4 : 184 |
| Anther | 5 : 107 | 6 : 147 | 7 : 131 | 8 : 175 |
| Leaf | 9 : 61 | 13 : 61 | 17 : 62 | 21 : 71 |
| Silique | 10 : 34 | 14 : 40 | 18 : 71 | 22 : 88 |
| Sepals | 11 : 42 | 15 : 48 | 19 : 72 | 23 : 75 |
| Internode | 12 : 43 | 16 : 43 | 20 : 70 | 24 : 76 |
| Root | 25 : 89 | 26 : 113 | 27 : 127 | 28 : 131 |

Supplementary Figure S4. Quantification of red intensity of RUBY expressing *Arabidopsis*. Figure 3A was converted to CIELAB color space and the a* axis was extracted and presented here in grey scale. Lower values in a* axis are greener while larger values are more magenta. ROI are highlighted in yellow, and the corresponding mean intensity of each ROI is shown in the table below.


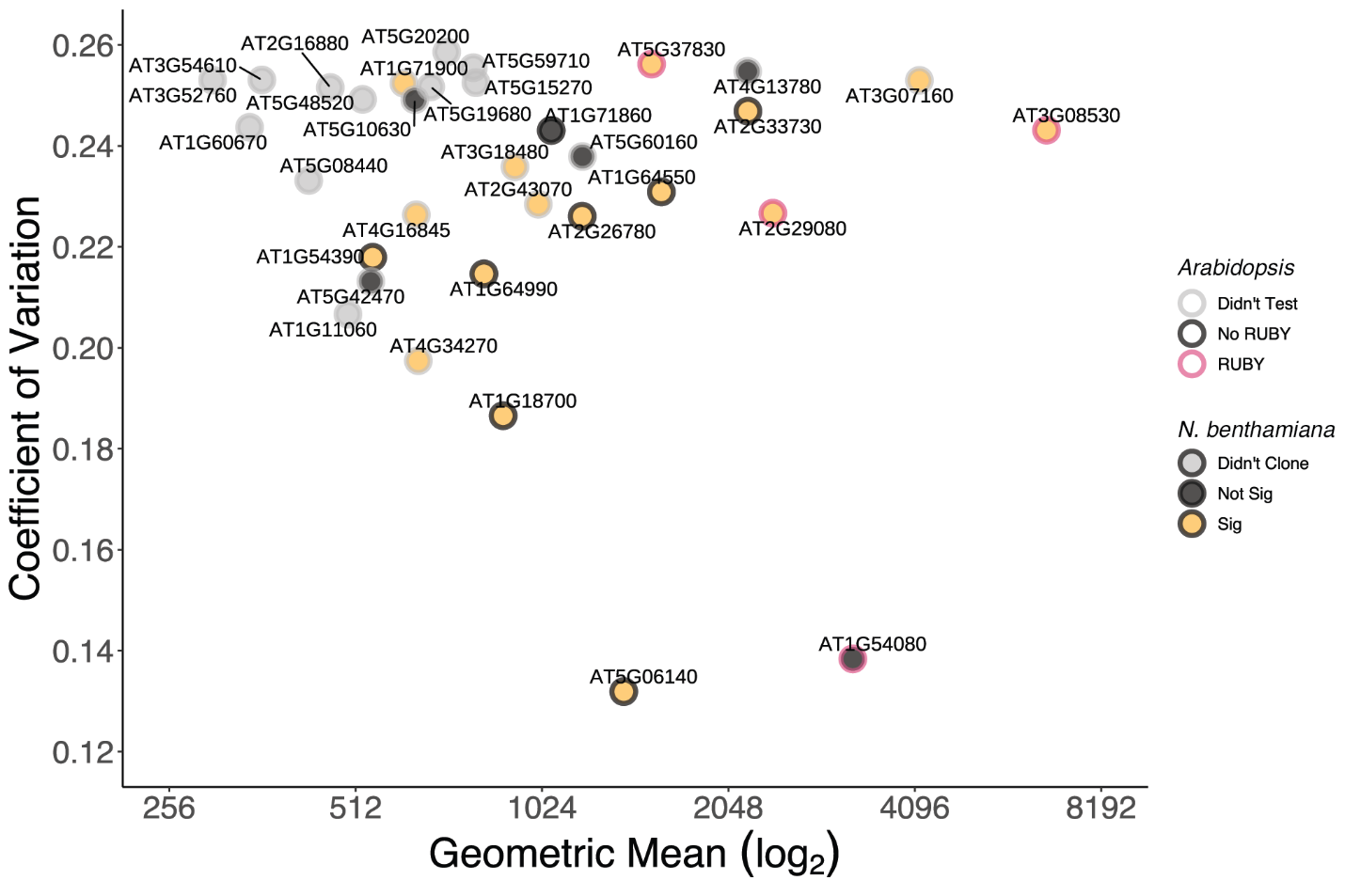


Supplementary Figure S5. Final 33 candidates and the summary of experimental results. All final 33 candidates that passed through the pipeline were shown. Y-axis is the coefficient of variation, and the x-axis is the geometric mean on a log base-2 scale. The points were colored in grey if the construct wasn’t cloned, black if the construct was cloned but showed no significant difference from negative control in transient *N. benthamiana* infiltration experiments, or yellow if the injection was significantly different from control. The points were outlined in grey if the RUBY construct wasn’t tested in *Arabidopsis*, black if the promoter did not give visible RUBY expression, and red if at least one part of the tissue displayed visible RUBY expression.


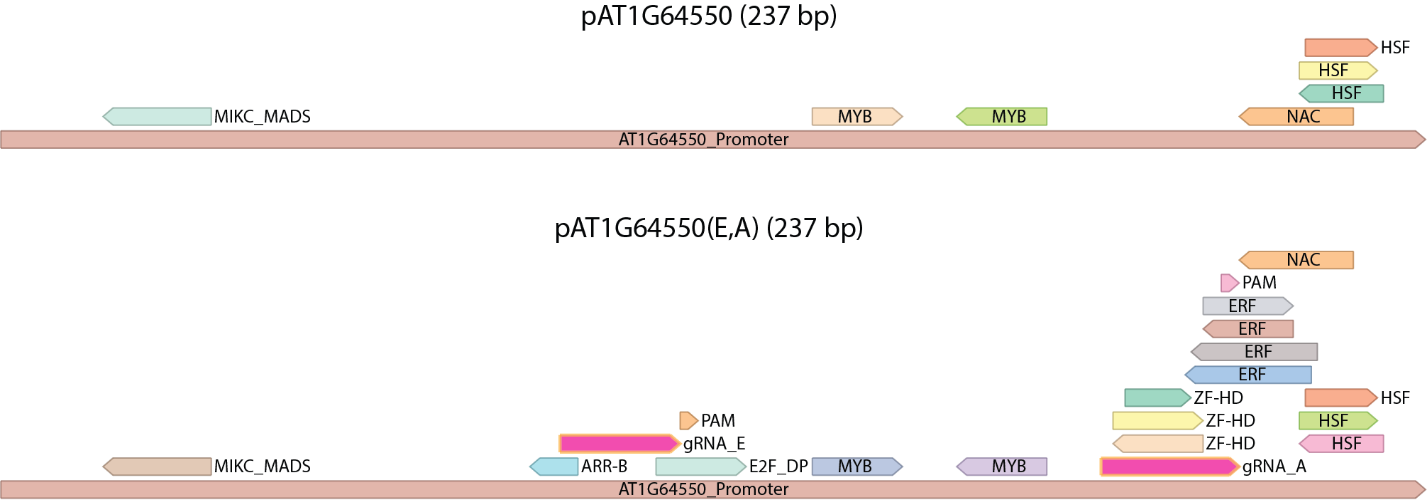


Supplementary Figure S6. The introduction of gRNA_E and gRNA_A target-sites (highlighted in pink) in pAT1G64550 did not disrupt any predicted motifs but introduced additional ones.

Supplementary Table S7

| **SRR** | **Sample Description** | **AT1G54080** | **AT5G37830** | **AT1G64550** | **AT2G29080** | **AT1G13320 (PP2AA3)** |
| --- | --- | --- | --- | --- | --- | --- |
| SRR3581336 | Seedling Hypocotyl (S.H) | 2791 | 1880 | 1294 | 1711 | 924 |
| SRR3581345 | Seedling Cotyledons (S.C) | 2906 | 2139 | 1782 | 3002 | 486 |
| SRR3581346 | Seedling Meristem (S.M) | 2703 | 1103 | 1849 | 2449 | 1.15 |
| SRR3581347 | Seedling Root (S.R) | 2434 | 1982 | 2097 | 2827 | 628 |
| SRR3581740 | Seedling Hypocotyl (S.H) | 2844 | 2202 | 1354 | 1958 | 552 |
| SRR3581831 | Seedling Meristem (S.M) | 1186 | 1139 | 1103 | 2310 | 776 |
| SRR3581833 | Seedling Cotyledons (S.C) | 2890 | 1853 | 1829 | 2682 | 246 |
| SRR3581834 | Seedling Root (S.R) | 2158 | 2012 | 2566 | 3070 | 610 |
| Mean | | 2489 | 1788 | 1734 | 2501 | 527 |

Values correspond to normalized read count.

Supplementary Table S8. Guide Sequences

| gRNA | Guide Sequence + PAM | Source |
| --- | --- | --- |
| A | GCAAAGGTGATTAACTGCAAAGG | (Bao et al., 2017) |
| B | AAAGGGGAAAAGAGTATTGGTGG | (Dahlman et al., 2015) |
| C | GGCAAGGCTGGCCAACCCATGGG | (Dahlman et al., 2015) |
| D | ACCCTGGCGGAGCTGATGGGTGG | (Dahlman et al., 2015) |
| E | TCTCAAGCTAGACTCTAGTGAGG | (Dahlman et al., 2015) |
| F | CATTGCCATACACCTTGAGGTGG | (Gander et al., 2017) |
| G | GTGGTAACTTGCTCCATGTCTGG | (Gander et al., 2017) |
| H | CTTTACGTATAGGTTTAGAGTGG | (Gander et al., 2017) |
| I | GAAGTCAGTTGACAGAGTCGTGG | (Gander et al., 2017) |


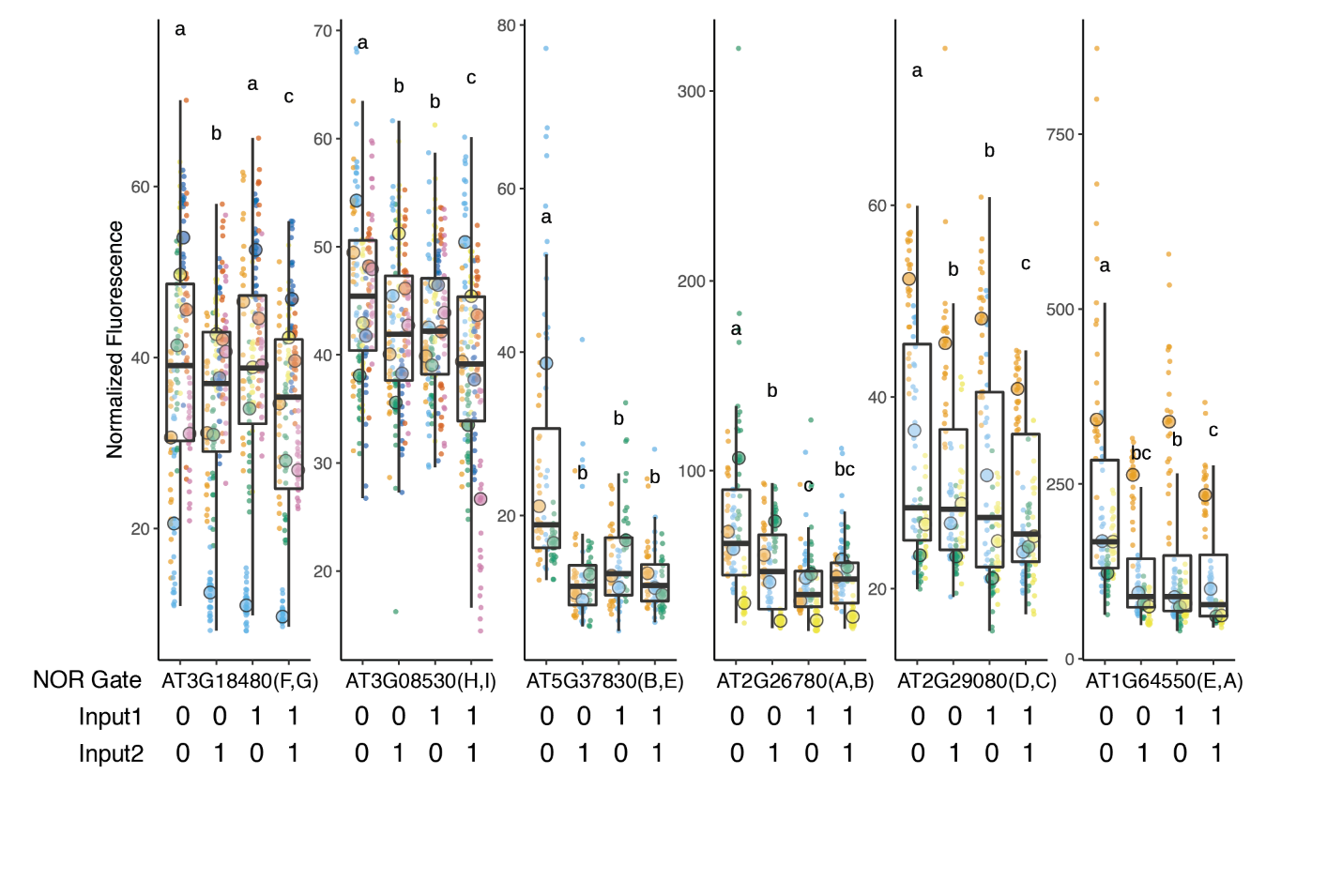


Supplementary Figure S9. Normalized Repression Data. Each biological replicate represented by a beeswarm plot and their median is marked by the large circle. The boxplot represents all the replicates together. The y-axis is normalized fluorescence by having the mPromoter:NLS_YFP signal divided by pUBQ10:NLS_mTURQ signal within the same construct. Each input for a given condition can be either ON (1) or OFF (0), and each NOR gate can accept four possible combinations of the two inputs.

Supplementary Table S10. List of primers used in the experiment.

Supplementary Table S11. List of plasmids used in the experiments, plasmid names correspond to Genbank files in Supplementary Data S11.

Supplementary Data S12. All the scripts used in the experiment.

Supplementary Data S13. All the plasmid maps of used in the experiment in Genbank format.

References

Bao, Z., Jain, S., Jaroenpuntaruk, V., & Zhao, H. (2017). Orthogonal Genetic Regulation in Human Cells Using Chemically Induced CRISPR/Cas9 Activators. *ACS Synthetic Biology*, *6*(4), 686–693. https://doi.org/10.1021/acssynbio.6b00313

Dahlman, J. E., Abudayyeh, O. O., Joung, J., Gootenberg, J. S., Zhang, F., & Konermann, S. (2015). Orthogonal gene knock out and activation with a catalytically active Cas9 nuclease. *Nature Biotechnology*, *33*(11), 1159–1161. https://doi.org/10.1038/nbt.3390

Gander, M. W., Vrana, J. D., Voje, W. E., Carothers, J. M., & Klavins, E. (2017). Digital logic circuits in yeast with CRISPR-dCas9 NOR gates. *Nature Communications*, *8*(1), Article 1. https://doi.org/10.1038/ncomms15459

Tokizawa, M., Kusunoki, K., Koyama, H., Kurotani, A., Sakurai, T., Suzuki, Y., Sakamoto, T., Kurata, T., & Yamamoto, Y. Y. (2017). Identification of Arabidopsis genic and non-genic promoters by paired-end sequencing of TSS tags. *The Plant Journal: For Cell and Molecular Biology*, *90*(3), 587–605. https://doi.org/10.1111/tpj.13511
